# Computational models of migration modes improve our understanding of metastasis

**DOI:** 10.1063/5.0023748

**Published:** 2020-11-05

**Authors:** Gabriel Shatkin, Benjamin Yeoman, Katherine Birmingham, Parag Katira, Adam J. Engler

**Affiliations:** 1 Department of Bioengineering, University of California, San Diego, La Jolla, California 92093, USA; 2 Department of Mechanical Engineering, San Diego State University, San Diego, California 92182, USA; 3 Computational Sciences Research Center, San Diego State University, San Diego, California 92182, USA; 4 Sanford Consortium for Regenerative Medicine, La Jolla, California 92037, USA

## Abstract

Tumor cells migrate through changing microenvironments of diseased and healthy tissue, making their migration particularly challenging to describe. To better understand this process, computational models have been developed for both the ameboid and mesenchymal modes of cell migration. Here, we review various approaches that have been used to account for the physical environment's effect on cell migration in computational models, with a focus on their application to understanding cancer metastasis and the related phenomenon of durotaxis. We then discuss how mesenchymal migration models typically simulate complex cell–extracellular matrix (ECM) interactions, while ameboid migration models use a cell-focused approach that largely ignores ECM when not acting as a physical barrier. This approach greatly simplifies or ignores the mechanosensing ability of ameboid migrating cells and should be reevaluated in future models. We conclude by describing future model elements that have not been included to date but would enhance model accuracy.

## I. INTRODUCTION

Cell migration is an integral part of many biological functions and pathological conditions, from immune response and wound healing to organ development and cancer metastasis. A cell's ability to move through space and reach its destination is critically important for it to fulfill its intended function. Depending on the cell type and the circumstances it finds itself in, cells can adopt different modes of migration,^1,2^ but all modes of migration can be described with the same basic steps: membrane extension, attachment formation, contraction, and rear release.^3^ Mechanisms that control each step and the degree to which each step affects migration varies with cell migration mode. Although a continuum of possibilities exists between the extremes of migration modes, two main subsets of migration, ameboid and mesenchymal migration, are among the most described, especially in the context of—but not exclusive to—cancer metastasis.

**FIG. 1.**
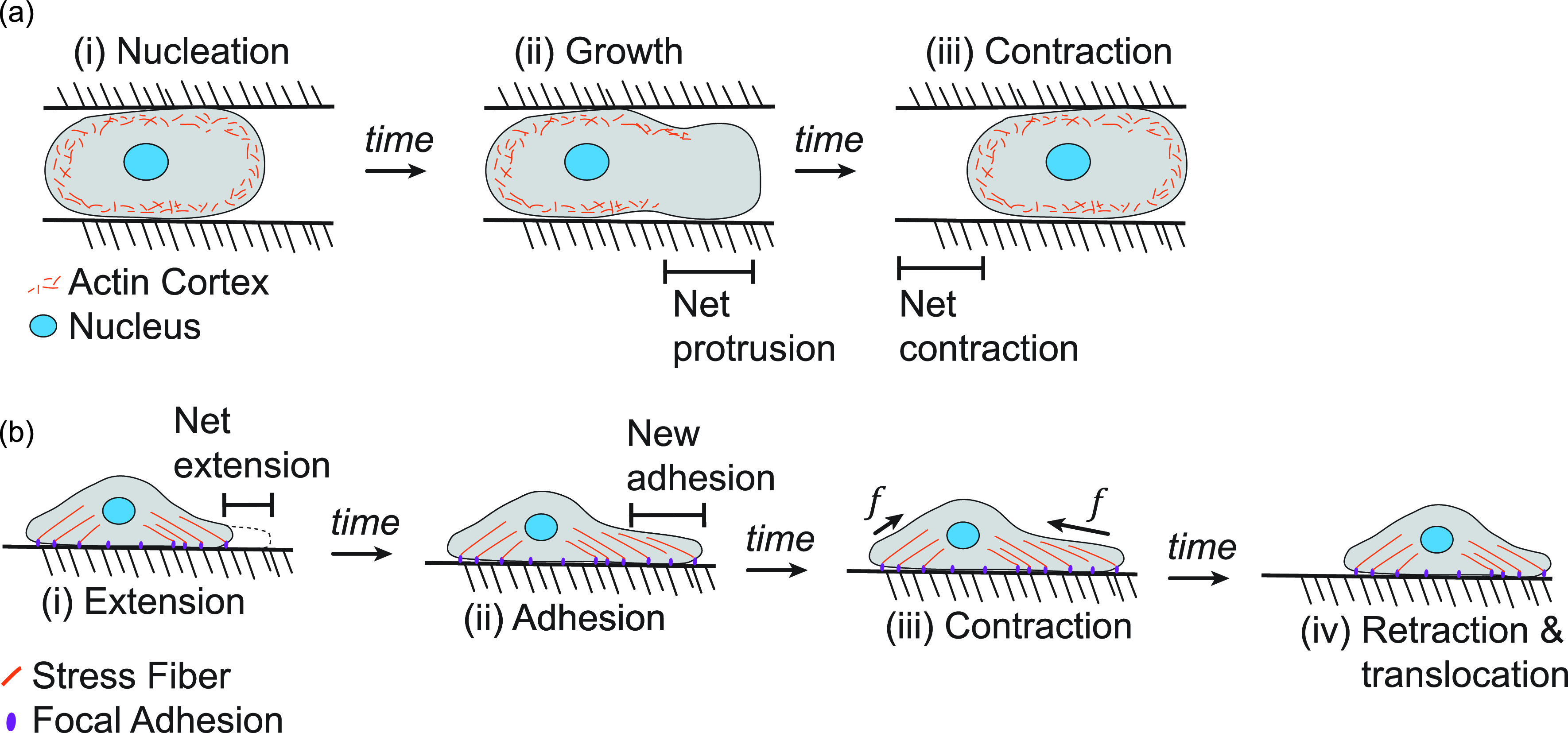
Processes of ameboid and mesenchymal migration in cancer. (a) Ameboid migration typically occurs as a three-step blebbing cycle with nucleation, growth, and contraction steps. (b) Mesenchymal migration typically involves a different process wherein cells extend their leading edge and adhere, contract, and release their trailing edge.

Ameboid migration occurs both in single-celled organisms, such as the ameba *Dictyostelium discoideum*, and within specific cell types in multicellular organisms, such as neutrophils.^4^ Cells undergoing ameboid migration exhibit rounded protrusions, i.e., blebs, and show little spreading on their substrate. This mode of migration progresses through a three-step blebbing cycle: nucleation, growth, and contraction [Fig. 1(a)]. The formation and expansion of these blebs are driven by weaknesses in the actin cortex and cytoplasmic pressure differences that cause the cellular membrane to expand outward.^5^ During the nucleation and growth phases, it is not clear to what extent the actin cortex ruptures, but there is a clear separation between the two.^6^ As blebs transition from growth to contraction, myosin causes the bleb to retract back into the main body of the cell, which can result in an overall movement of the cell toward the direction of the bleb expansion.^7^ It should be noted that ameboid cells can exhibit other types of protrusions that are closer to the mesenchymal end of the migration spectrum, i.e., pseudopods.^8^ Pseudopods initiate as blebs; however, their expansion from the cell body is coupled with continuous active expansion of F-actin in the underlying cellular membrane. Both types of protrusions can form on the same ameboid cell, and blebs and pseudopods have been shown to operate cooperatively during chemotaxis.^9^ Regardless of the main protrusion type, ameboid cells exhibit clearly defined polarization with a leading and trailing side.^10^ They can also travel at relatively fast speeds compared to other modes of migration (∼10 *μ*m/min)^11^ depending on their surroundings. Additionally, these cells do not form strong focal adhesions with the surrounding extracellular matrix (ECM)^4^ and tend to be more processive in their migration, especially in the dense matrix.^12^

Conversely, a wide variety of migrating cells contract against focal adhesions in protrusions from the main cell body to move in a second method often termed mesenchymal migration [Fig. 1(b)]. Protrusions used in this mode are typically classified as filopodial or lamellipodial, with the former being thin spindle-like protrusions and the latter being sheet-like protrusions.^3^ Additionally, cells in the mesenchymal mode form strong focal adhesions to the ECM^13–15^ and, as a result, appear to spread over their substrate; this mode is typically slower than ameboid migration^4^ and is classically observed on two-dimensional substrates. In addition to morphological differences, there are mechanistic differences between the two modes of migration; cytoplasmic pressure gradients drive ameboid migration^12^ often in confined settings, whereas mesenchymal migration is driven by actin polymerization and the active maturation and turnover of focal adhesions coupled with actin-myosin contraction^16^ across a more spread cell. This different mechanism is not entirely distinct from the pressure-driven flowing actin networks of ameboid migration; mesenchymal migrating cells exhibit a retrograde actin flow away from the leading edge and toward the main cell body.^17,18^ However, computational models typically treat different migration modes as entirely distinct for the sake of simplicity and are used to answer specific questions. Contact guidance between these modes is also markedly different and results in proliferative differences that could underlie migration^19^ and hybrid cell formation.^20^ A summary of major differences in migration outcomes^21–30^ is shown in Table I.

**TABLE I.** Major performance differences between migration modes for cancer cells.

	Ameboid	Mesenchymal	Reference
Migration speed	2–25 *μ*m/min	0.1–1 *μ*m/min	21–23
Persistence	Low	High	24, 25
Morphology	Rounded	Elongated	23, 26
ECM attachment	Weak, short term, and lower integrin expression	Integrin clusters forming focal adhesions	23, 27
Migration in ECM	Squeezing or blebbing through ECM pores	Adhesion-mediated tractions and ECM degradation	23, 28, 29
CSK organization	Actin cortex	Actin meshwork, contractile stress fibers, and microtubules	23, 29, 30

Although these two extreme modes represent a majority of cellular movements observed *in vivo* and *in vitro* (and models describing mesenchymal migration are significantly more common than ameboid migration), several other modes, both intermediate and distinct, have also been described but were omitted here for clarity.^31^ The use of these modes often depends on the environment's dimensionality (which can regulate adhesion assembly^32^), on the cell type, and on the receptor-ligand pairs as with selectins used in leukocyte migration.^33^ These modes often exhibit distinct features, making them easily identifiable, such as the crescent moon shape and gliding motion of keratocytes,^34^ but exist in a continuum between mesenchymal and ameboid modes.

## II. MIGRATION AND CANCER METASTASIS

Cancer is the second leading cause of death in the United States, and the vast majority of its mortality is associated with secondary tumor formation.^35^ In order for cancer cells to metastasize and form secondary disease, they must migrate out of the primary tumor, intravasate into the bloodstream, and then extravasate into other tissues throughout the body.^36^ Cells within tumors are also very heterogeneous, making it difficult to separate indolent cancers from deadly ones, as only a subset of cells is able to disseminate from the main tumor and the others remain stationary and benign. Alongside migration mode, directionality is incredibly important for metastasis, yet remains poorly understood in certain contexts. For example, cancer cell chemotaxis (i.e., migration along a chemical concentration gradient) has been studied in-depth in ameboid cells but comparatively little for mesenchymal cells.^4,37^ More recently, effort has been made to understand the effect of cells' mechanosensing on migration. For example, the progression of metastatic breast cancer has been related to the levels of mechanosensing proteins in stiff ECM.^38^ Cells migrate at different speeds depending on substrate stiffness and oftentimes exhibit durotaxis, the ability to sense and migrate up a stiffness gradient.^39–41^ However, this seems counterintuitive for understanding cancer metastasis, as often times, the tumor microenvironment becomes much stiffer than the surrounding healthy stroma due to matrix secretion and cross-linking by cancer-associated fibroblasts.^42,43^ In these cases, the metastatic cells must exhibit adurotactic behavior in order to leave the primary tumor, which further complicates our current understanding of cancer cell migration and metastasis. Adding yet another level of complexity is the observation that tumor cells migrate in both the ameboid and mesenchymal modes and, depending on their environment, can switch between the two.^1,2,44^ They can also migrate individually or collectively,^45^ and their migration is highly dependent on the physical properties of their niche, such as stiffness, porosity, dimensionality, and toporgaphy,^46^ which can change as a result of clinical care.^47^ Despite these many influences, tumor migration models, thus far, largely focus on intracellular mechanisms governing mesenchymal and ameboid modes, and thus, we will describe the effects of additional modes and matrix properties in the context of model limitations later.

## III. COMPUTATIONAL MODELING OF MIGRATION

Cancer cell interactions are often very complex; reductionist approaches using model systems, e.g., microfluidic bioreactors,^48^ explore many isolated variables, and more complex models may even include the vasculature to study extravasation.^48,49^ However, despite the simplicity of these model systems, fidelity with *in vivo* disease progression may be limited or at least require context and necessitate significant engineering to generate robust datasets. Computational models, however, may offer an alternative—where applicable—to create and test reasonably complex niches *in silico* to understand migration mechanisms prior to experimental studies, thus better informing the design of more effective and efficient experimental studies.

**FIG. 2.**
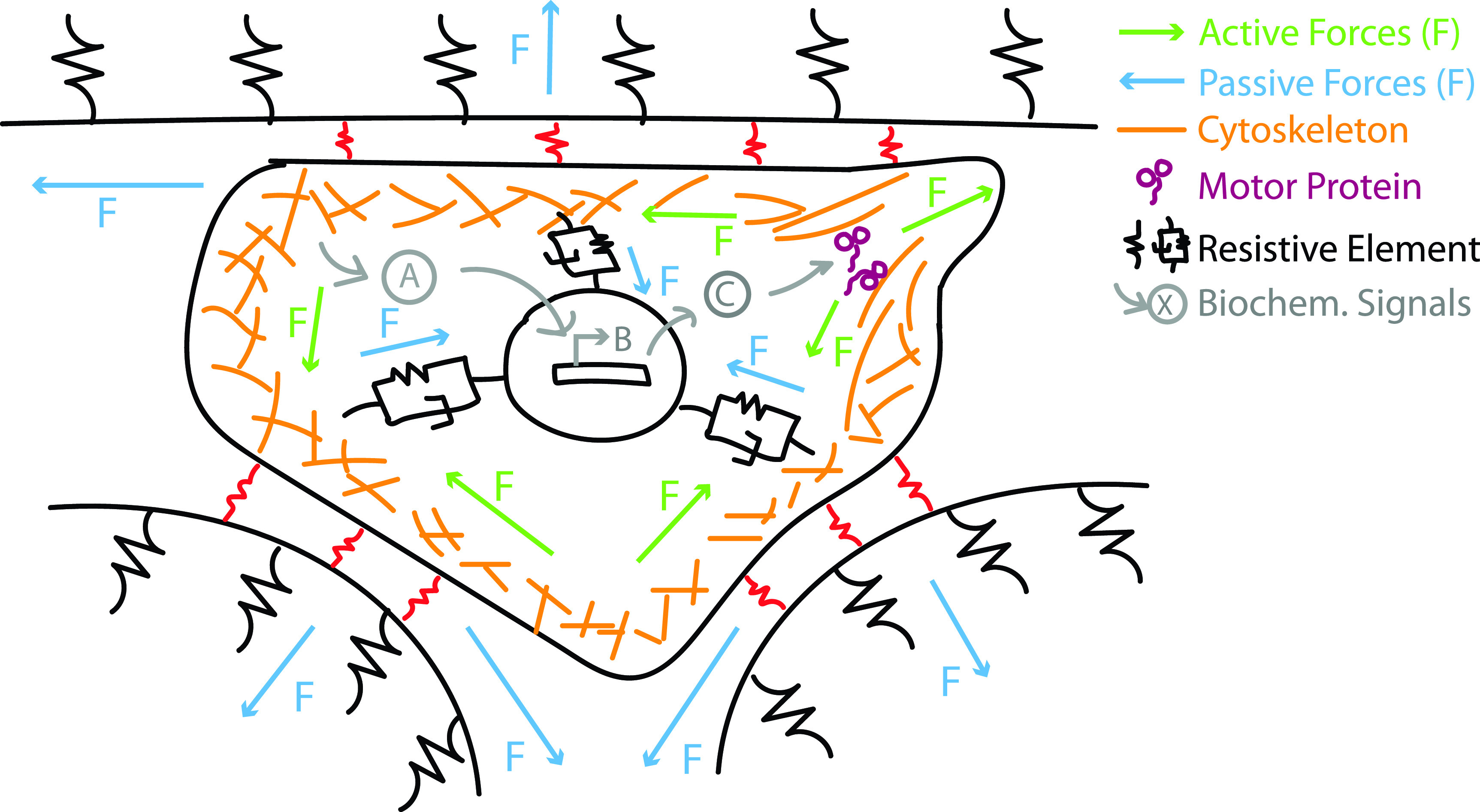
Conserved components of computational models of cancer migration. Four concepts are typically present to some degree in computational models of migration: force balance, mass conservation, active forces, and passive forces. Each is illustrated here and where active forces are those generated by motor proteins and polymerization and depolymerization of cytoskeletal filaments and passive forces are those from the viscoelastic parts of the cytoskeleton and ECM as well as from molecular friction.

A key consideration for any computational model is the complexity of its physics; over- or under-determined systems can limit applicability and predictive value. With respect to cell migration, many models consider the following key concepts: force balance, mass conservation, biochemical activity, active forces, and passive forces^50^ (Fig. 2). Force balances are used in all models to determine the net force magnitude and direction, which governs a cell's movement. Mass conservation is especially prevalent in models with a focus on protrusion dynamics or morphology changes in migrating cells to determine cells' changing shapes with a constant mass. Biochemical activity connects intra, inter, and extracellular signaling to cellular and extracellular mechanics. Active forces include forces generated by cytoskeletal dynamics such as actin and microtubule polymerization and depolymerization and actomyosin contractility. On the other hand, passive forces include reaction forces arising within cells, between neighboring cells, and between cells and the surrounding environment elastic strain, viscous drag, and molecular friction.^50^ The integration of these components in the model, the degree to which they affect each other, and, more broadly, overall migration depends on a number of intracellular and extracellular parameters in the model's framework. A brief overview of some of the modeling approaches discussed below,^51–68^ and associated equations, is shown in Table II. Note that this is meant to introduce readers to the various ways that physical laws governing cell migration can be described mathematically and is meant to direct readers to specific examples where these methods are applied. As computational costs continue to go down, the equations and models can become more detailed and combine multiple approaches into hybrid models. For the remainder of this review, we will broadly discuss how the above described key concepts are modeled and affect cell migration mode citing specific examples.

**TABLE II.** List of frequently physical frameworks used to model cancer cell migration and their applications.

Common modeling approaches	Applications and examples
Chemo-mechanical models based on force-dependent reaction kinetics.	Used to model sub-cellular processes such as cell-substrate bond formation, filament polymerization and gliding and mechanosensing-based changes to predict resulting cell adhesion, traction, and migration (e.g., various spring/dashpot models,^51–53^ active matter models,^54^ and molecular clutch models^55^).
$F\left(\mu \right)-\gamma \left(F,n\right)v=0$… Force balance between active forces driven by chemical potential, μ, and dissipative forces, γv.	
$\gamma \left(F,n\right)=K\tau_{0}e\mathrm{xp}(-\frac{F}{{nF}_{0}})$… stiffness, K, and number, n, and kinetics of molecular bonds, $\tau_{0}$ dictate drag coefficient, γ.	
Agent-based models focusing on force balance between individual cells and their environment.	Used to model cell populations interacting with each other and the environment. Coarse grained to implicitly include effects of various sub-cellular processes (e.g., force-based models,^56–58^ energy-based models,^59,60^ and lattice-based/cellular Potts models^61–63^).
${\vec{F}}_{\mathrm{active}}+{\vec{F}}_{\mathrm{passive}}+{\vec{F}}_{\mathrm{dissipative}}=0$…	
or an energy minimization approach	
$E=\sum \lambda_{i}{\left(A_{i}-A_{0}\right)}^{2}+\sum \sigma_{ij}l_{ij}+\sum \frac{dU}{d{\vec{r}}_{i}}.{\vec{r}}_{i}$	
Thermodynamic models based on equilibrium and non-equilibrium work-free energy change relationships	Used to model both cellular and sub-cellular processes and assess the energetic states that the system can occupy (e.g., free-energy-based models^64,65^).
$\Delta F=\sum \Delta \mu_{i}-k_{B}T\ln\frac{\Omega }{\Omega_{0}}$…Free energy change of the system	
$W=\int F_{\mathrm{active}}dx$… work done by the system	
Equilibrium… minimize (ΔF)	
Non-equilibrium … ΔF≳W	
Continuum phase-field models	Used to describe cell and surrounding free space as an evolving phase-field, with the moving boundary representing the cell membrane. Well suited to describe collective migration^66,67^ and migration of cell monolayers.^68^
$\frac{d\Phi_{i}}{dt}+{\vec{v}}_{i}\cdot \vec{\nabla }\Phi_{i}+\frac{\delta F}{\delta \Phi_{i}}=0$… Describes the dynamics of the cell shape in response to free energy changes. The free energy functional, F, is chosen so that minima correspond to phases (i.e., intracellular and extracellular environment) of the system.	

## IV. MESENCHYMAL MIGRATION MODELS: APPLICATIONS AND DIRECTIONS

Computational models describing mesenchymal cell migration primarily focus on intracellular active forces driving protrusion and retraction of lamellipodia and filopodia, balancing these forces against elastic, viscous, and friction forces within and outside the cell, and mass balance that defines the cell shape as the cell migrates under the action of these forces.^50,69–71^ At the nanoscale, these models can focus on the dynamics of actin polymerization and depolymerization, force generation by individual myosin motors, binding and unbinding of adhesion receptors to the extracellular matrix, clustering of adhesion receptors and maturation of adhesion sites, and binding of polymerized actin filaments to these receptors to form adhesion complexes.^27,72,73^ At this scale, models incorporate force generation and sensing aspects such as conformational changes in adhesion complex proteins, recruitment of additional actin-myosin fibers, and branching of actin fibers. At the mesoscale, models focus on cell spreading, filopodial and lamellipodial protrusion and contraction, coupling between the nucleus and the cytoskeleton, viscoelastic strains within the cytoskeleton and the nucleus under the influence of active and passive migration forces, and resulting cell shape changes.^30,61,70,74,75^ Models at this scale are particularly useful for predicting cell shape dynamics and interactions between two neighboring cells or a single cell and its environment. At the microscale, the focus of modeling is on overall cell migration dynamics under the influence of a driving force balanced by the drag forces from the environment.^56–58,76,77^ At this scale, the goal of the models is to predict cell migration velocity and path persistence as a function of the mechanistic interactions between the cell, its neighbors, and the surrounding extracellular matrix. Computational models may focus on a specific length scale or combine multiple length scales to predict migration dynamics. Models can also vary in their representation of the extracellular environment. For example, to describe cell migration on a 2D substrate, the ECM can be described as a continuous elastic material or discretized into a collection of binding sites connected to springs.^61,63,78–80^ In 3D, the ECM can be described as a viscous continuum, discretely as a collection of randomly or uniformly distributed binding sites, or as fibers distributed randomly or along the grid in 3D space.^56,62,76,81,82^ Depending on the choice of the ECM model, various aspects of cell–ECM interactions can be integrated such as ECM degradation, ECM remodeling, contact guidance along aligned fibers, and squeezing of cell through ECM pores. A common thread between all these models of mesenchymal cell migration is that the migration is driven by forces generated within long protrusions that grow along the surface in search of sufficient binding sites in 2D and along the length of fibers in 3D.

**FIG. 3.**
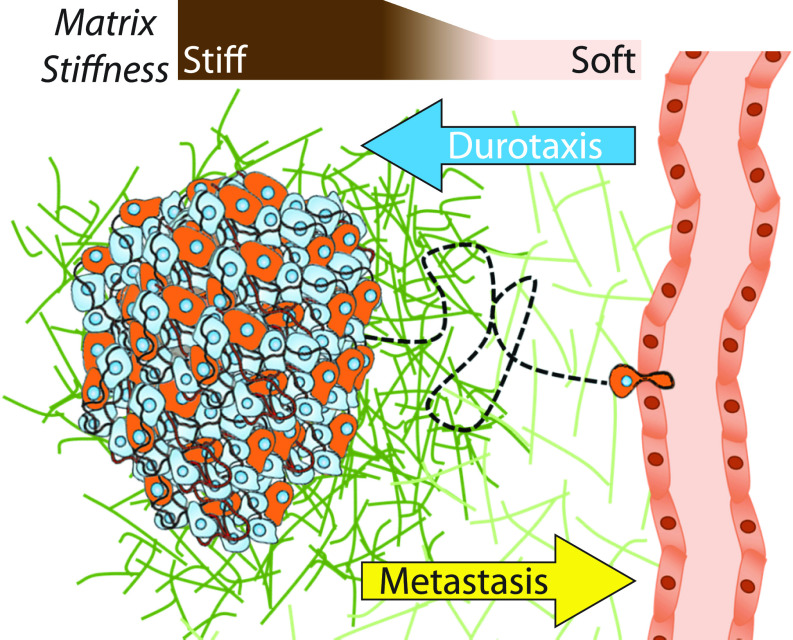
The migration paradox. Most cells migrate toward stiffer regions of tissues in a process called durotaxis.^59^ However, tumor cells must migrate from stiff tumors through the progressively softer matrix to disseminate from a tumor core and intravasate into the blood stream. This metastatic migratory process is counter to conventional thought on stiffness gradient migration, and it is not clear which migration mode, if any based on available data, permits such migration. Note that for simplicity, additional cell types, e.g., cancer associated fibroblasts, have been omitted but play a key role in niche remodeling nonetheless.

The primary goal of these computational models has been to predict how fast and persistently cells will migrate along or within a given substrate depending on their specific mechanical and chemical properties. Models have also been successful in predicting experimentally observed behavior of the biphasic dependence of migration speed on ECM density, adhesivity, and stiffness.^41^ Models can also recreate qualitative trends in migration persistence, which have been observed experimentally and clinically. More recently, modeling of experimentally observed emergent phenomena such as chemotaxis, durotaxis, haptotaxis, and contact guidance, which direct cells along specific directions, is gaining attention. How mechanobiology affects migration, i.e., how do changes in niche parameters direct processive migration and ultimately intravasation, is of extreme importance in understanding processes such as wound healing and cancer metastasis. Most adherent cells migrate toward a stiffer region of a substrate when presented with a gradient,^83^ i.e., “durotax;” yet, tumors are inherently stiff relative to adjacent, soft stroma *in vivo*^84,85^ created by cancer-associated fibroblasts. This creates a cancer cell “migration paradox” where tumor cells must migrate down ECM stiffness gradients that otherwise support migration in the opposing direction (Fig. 3). Here, we will focus on a few mesenchymal cell migration models that explain cancer cell durotaxis and their applicability toward answering the cancer cell migration paradox. That being said, it is important to note that we are restricting our discussion and this concept to the disseminating cells from the tumor. Many other cells must migrate toward the tumor, i.e., durotax, including inflammatory cells, among others.

To start, many models describe migrating cells as polarized with a higher probability of protrusion at the leading edge and retraction fibers at the trailing edge. Focal adhesions form preferentially along the leading edge and dissociate more frequently along the trailing edge, resulting in net motion in the direction of the leading edge.^3^ Some computational models replicate durotaxis on a surface using polarized cell filopodia, which are more likely to grow along elastic fibers aligned along an increasing stiffness gradient.^81^ This approach to simulating migration succeeds in predicting the general movement of durotactic cells and in creating a more realistic representation of ECM fiber networks rather than conventional models that treat the substrate as a continuum. Yet, such a rigid integration of durotaxis is not without disadvantages, e.g., durotactic behavior has to be built into the model, rather than durotactic behavior arising from it. Furthermore, while the elastic fibers can be deformed, the cell cannot remodel or degrade them. Thus, from such polarity-based models and those that take similar approaches, e.g., adhesions that bind more strongly on the stiffer region than softer regions forcing polarized shapes a priori,^86^ it may be difficult to infer mechanisms of durotaxis or even adurotaxis as observed in cancer *in vivo*. These models are, however, convenient to study the effect of inter-cellular heterogeneity in these parameters on overall population dynamics when predicting collective cell behavior. Indeed, within a tumor, not all cells metastasize, and thus, cancer migration models should highlight both cellular and temporal heterogeneity^87^ when describing gradients.

One of the early models that explained the origin of durotactic behavior rather than making it an intrinsic property of migrating cells was by Schwarz *et al.*, which used a simple 2 spring attachment-detachment model between the actin myosin force generating elements, cell membrane attached adhesion protein, and the substrate.^51^ The model showed that as the substrate stiffness increases, the rate at which the force is generated within the actin–myosin filament increases, leading to larger overall traction forces within the lifetime of a cell–substrate adhesion bond. The increase in traction forces on stiffer substrates drives cell migration up the stiffness gradient explaining durotaxis. This phenomenon has since been integrated as an *a priori* mechanism of cell migration in a number of other models. A more recent addition to models explaining durotaxis is based on a rigidity sensing-based change in the biochemical activity of motor protein regulating units within the cell.^72^ Increased feedback from a stiffer substrate drives increased myosin activity and higher speeds for cells in stiffer regions, driving an accumulation of cells up a stiffness gradient. A third alternate model focuses on the mechanical response of the ECM fibers rather than that of the cell itself. The model is based on fiber mechanics that suggests that the deformation/extension of a fiber decreases as a cell gets closer to the point where the fiber is crosslinked to a stiffer environment. This generates a stronger passive force on the cell, pulling the cell toward the stiffer regions and driving durotaxis.^81^ There are many other models that explain durotaxis through variations or combinations of the above described themes that we are not discussing here. A more recent model by Heck *et al.*^88^ suggests yet another possible explanation for durotactic behavior. This model accounts for ECM as an obstacle that the cell must negotiate or degrade; this type of confined migration is often observed in dense tissue where degradation by the cancer cell proceeds filopodial extension^89^ and requires significant deformation (which can be measured in high throughput with fluidics^90^). The model predicts that migration is most affected by ECM stiffness, cell adhesion strength, and protrusion properties, e.g., number, lifetime, and length. The model shows the standard correlation between matrix stiffness and migration speed, consistent with past models and experimental observation using degradation and filopodial extension.^91^ They also show that adhesion turnover makes migration more processive. By incorporating force-dependent adhesion turnover rates and increased forces in stiffer ECM regions, they show an additional mechanism for durotactic behavior driven by increased persistence. A connection between processive migration and adhesion strength is also supported experimentally as seen when a weakly adherent phenotype results in a reduced disease-free interval.^92^ The implication that protrusion activity and matrix deformation in three-dimensions, not the development of robust focal adhesions as in two dimensions, as the origin of their processivity, may be consistent with some results in three dimensions.^28^

The models discussed so far cover most known aspects of mesenchymal cell migration and also can be used to describe a number of emergent phenomena. With regard to durotactic behavior, they all suggest using one argument or another that cells should durotax, and for the most part, adherent cells do so. However, the cancer cell migration paradox of metastatic cells migrating against a stiffness gradient to metastasize still remains unanswered. It may be necessary to develop mechanistic models that explain anti-, a-, and durotactic migration that focus not on the ECM but rather on the cytoskeletal elements that drive mesenchymal migration; here, we summarize two such models. To interrogate such cytoskeletal elements, the most common mesenchymal model used has been the molecular motor-clutch,^93^ which employs multiple molecular motors to pull actin filaments toward the cell body on a compliant, continuous substrate; conversely, the molecular clutch binds to actin stochastically and links it to the extracellular environment via a force-dependent Bell model connection.^94^ These models typically predict a stiffness-dependent relationship for migration where on compliant substrates, the motors undergo load-fail cycles, whereas on stiff substrates, the complex slips. This model predicts biphasic behaviors in force and migration that can reinforce adhesion,^95^ with an optimal stiffness region that cells will most likely migrate to. The model, thus, does not always predict durotaxis or antidurotaxis, but rather migration in the direction of the preferred stiffness for a given cell type. Optimal stiffness for a cell type is dependent on the actomyosin contractile force that the cells can generate and the number of clutches between the cell and the substrate. Overall, the clutch model may provide a possible reason for the adurotactic migration of metastatic cancer cells away from a stiff tumor ECM, but validation of experimentally observation of adurotaxis by highly metastatic cancer cells needs to be further explored. Another model in which the mechanical environment can be considered in migration is an equilibrium thermodynamics model. This model provides an alternative way to characterize the two-way feedback loop between cell contractility and matrix realignment. This model calculates the total change in free energy of the system, consisting of the energies of the cell, matrix, and adhesions to determine whether or not migration will occur as the system tries to move into a lower energy state.^65^ Similar to the clutch model, this thermodynamic migration model predicts a biphasic migration response to matrix stiffness and depends on the contractile force that a cell can generate and the strength of its adhesions. It is possible to envision that in the presence of gradients, cells could adurotactically migrate to an optimum away from the tumor, thus achieving goals similar to the clutch model.

Overall, due to important fluctuations in force,^96^ heterogeneous adhesion within a tumor,^92^ and stiffness gradients (vs changing but static substrates in these models) on the stroma,^85^ there is a need for new models where these or similar parameters are incorporated together, e.g., maximum force generated by a stress fiber, catch bond dynamics, etc. Changes to the force-bond lifetime relationship could then result in anti-, a-, and durotactic behavior depending on how each parameter varies with the others.

## V. AMEBOID MIGRATION MODELS

Ameboid migration is dominated by propulsive membrane blebbing, i.e., the key concept of force balances, rather than spreading and forming strong focal adhesions to their substrate, i.e., the key concept of active contractile forces.^4^ It also relies less on modification or degradation of the adjacent ECM and more so on becoming highly deformable and pushing through matrix pores. Despite the differences between mesenchymal and ameboid migration, cancer cells display unique plasticity in their ability to switch between modes,^24,97^ making our understanding of ameboid migration even more critical. Unlike mesenchymal migration, ameboid migration models reviewed here tend to focus on intracellular parameters, e.g., the development of pressure gradients to form blebs or propel the cell forward,^12^ rather than on the cells' physical environment. Despite this, ameboid cells may indirectly mechano-sense, which may be necessary for tumor metastasis.

A common characteristic of ameboid models is that they highlight a specific aspect of migration based on the nature of their model. For example, cell membrane deformability—modeled as a system of springs—has been used to determine a cancer cell's migration speed through confined spaces,^70^ such as pores in a matrix. This model suggests that, but does not assess, stiffness gradients are able to drive a cells' direction of travel, but polarization in this model is simply defined to guide the cells through the obstacles and is not a result of it. The ECM playing only a passive role in influencing cell behavior is also observed in an earlier model from the study by Lim *et al.*^7^ In this model, the cell has a permeable actin cortex inside an impermeable outer membrane, with adhesion points connecting the two. Cell movement occurs when membrane-cortex adhesions rupture, the outer membrane expands, and the actin cortex is pulled toward the rupture by a cytoplasmic pressure gradient; again, the matrix is only an obstacle and polarization is built in to the model rather than a result of it. From such models, neither mechanosensing nor migration from gradients has been considered.

Ameboid-like migration has been frequently observed in 1-^98^ and three dimensions^99,100^ when the environment requires less adhesive, confined migration distinct from mesenchymal modes. Despite not engaging traditional machinery, cancer cells in this mode and confinement do recognize substrate stiffness unlike the models mentioned previously, and it is observed that stiffer, confined spaces support more ameboid migration.^26^ Thus, models that highlight this behavior often consider possible ways that gradient sensing could occur in ameboid migration, either *de novo* or as a switch between models. For example, probabilistic models highlight that migration mode switching occurs in heterogenous matrix conditions and that both migration modes and plasticity are advantageous in heterogenous tumors because cells can sense their niche and switch modes as needed. Modulating the degree of cytoskeletal polymerization can also induce transitions as observed by Niculescu *et al.*^71^ Simulated cells displayed ameboid blebbing or a spread lamellipodium and gliding behavior simply by changing maximum actin polymerization. Each mode fed back on itself, and so switching events were rare as in *in vitro* observations but were not dependent on local conditions, i.e., cell could not sense environmental changes.

All models discussed in this section portray a common trend and limitation of computational models used for ameboid migration: focus remains on processes within the cell rather than interactions between the cell and its environment. Even for the few models that incorporate interactions between migrating cells and their physical environment, they still simplified as they restrict the cell from taking some shape or prevent the membrane from expanding into the physical obstacle. Although an emerging part of the literature,^100^ mechanosensing in this mode should be validated so that we better understand the environmental conditions and gradients that could result in migrating cells switching between modes^1,2^ and if ameboid migration could support adurotaxis as the mechanisms still remain to be elucidated (Fig. 3). While these models are able to analyze how cells change the shape and what sized spaces they can fit through, thus providing information about the process of migration, they are limited in that they only account for one portion of complicated processes like tumor metastasis and adurotaxis. Thus, critical questions remain: Under what circumstances do ameboid migrating cells stop durotaxing and move out of the stiffer tumor region? Do ameboid migrating cells respond differently to stiffness gradients than mesenchymal migrating cells? What takes precedence if ameboid migrating cells are presented with competing durotactic and chemotactic signals?

## VI. LIMITATIONS AND CONCLUSIONS

The models discussed in this review show varying degrees of importance given to cells' mechanosensing capabilities and the effects of the ECM on migration. This is especially important to understand in cancer, where cells' ability to sense and migrate against a stiffness gradient may contribute to their ability to metastasize.^38,42,43^ Due to the complex nature of cell migration and mechanosensing, computational models offer one of our best ways to understand and learn about migrating cell behavior. However, none of the models discussed consider all the variables involved in migration and to do so would likely be overcomplicated, computationally taxing, and, therefore, infeasible. Even the models that give extensive thought to the cells' physical environment do not consider cells' ability to switch between migration modes as other models do.^26^ Beyond this, few models explore the cooperative or inhibitory migration behaviors arising from interactions between multiple cell types such as metastatic cancer cells, cancer-associated fibroblasts, and senescent tumor cells, which can all occupy neighboring spaces within the tumor microenvironment. Multicellular interactions can be extremely complex and can assist or hinder durotactic behavior through short- and long-range mechanical and chemical coupling.^101–104^ No single modeling framework captures the complete breadth of these observations as far as we are aware. The current work on computational cell migration models has undoubtedly helped increase our knowledge of processes like cancer metastasis and migration in general to address the cancer cell migration paradox, but there are still many unanswered questions on how cell migration is guided by gradient sensing mechanisms illustrated in the paradox (Fig. 3).

Future models and experiments should examine the effects of stiffness gradients on ameboid migrating cells, despite their comparatively weak focal adhesions, and how the effects of the physical environment change as cells transition between the ameboid and mesenchymal migration modes. Additionally, the development of computational models and mechanistic explanations of migration are required in order to attain a balance between model accuracy, potential insight, and computational complexity. Mesenchymal migration models have shown significant inclusion of mechanosensing and the ECM's effects on migration, but they still omit other key parameters that could influence migration, such as fluid convection and flow,^105^ leaving room for growth in future models.

## Data Availability

Data sharing is not applicable to this article as no new data were created or analyzed in this study.
